# Radial Artery Pseudoaneurysm Following Cardiac Catheterization: A Case Report

**DOI:** 10.7759/cureus.19284

**Published:** 2021-11-05

**Authors:** Binayendu Prakash, Sandipan Mukhopadhyay, Pankaj Singodia, Mandar M Shah

**Affiliations:** 1 Department of Cardiology, Tata Main Hospital, Jamshedpur, IND; 2 Department of Radiology, Tata Main Hospital, Jamshedpur, IND; 3 Department of Plastic Surgery, Tata Main Hospital, Jamshedpur, IND

**Keywords:** punctures, coronary artery angiography, anticoagulants, artery pseudoaneurysm, cardiac catheterization, radial artery

## Abstract

The transradial approach is the most preferred method for cardiac catheterization. The radial route approach has many advantages, including fewer puncture site-related complications and early mobility and discharge. The vascular complications include radial artery spasms, occlusions, dissections, perforations, and compartment syndrome. Although pseudoaneurysms are a well-known complication of femoral access (0.2%-3%), pseudoaneurysms are very infrequent (0.05%) after radial artery access. Very few cases of radial pseudoaneurysms have been reported to date. We present a rare case of an 82-year-old man on dual antiplatelet and anticoagulant therapy who underwent coronary angiography via the radial route. The patient developed a pseudoaneurysm requiring surgical intervention.

## Introduction

The transradial approach for cardiac catheterization was first described in 1989. Coronary intervention through the radial approach is currently the most preferred method. The radial route has many advantages, including fewer complications related to the puncture site, less bleeding, and early mobility, and discharge. However, the radial approach is not free of complications. Rare vascular complications include radial artery spasms, occlusions, dissections, perforations, and compartment syndrome [1-4].

Although pseudoaneurysm is a well-known complication of femoral access (0.2 to 3%), this complication occurs very infrequently (0.05%) after radial artery access [5-6]. After the catheter or sheath is removed, a clot usually forms at the arteriotomy site, which seals the lumen and prevents the continuous outflow of blood. If the formation of the thrombus is not adequate, a pseudoaneurysm communicating with the arterial lumen can form.

To date, very few cases of radial pseudoaneurysms have been reported. We present a rare case of pseudoaneurysm that required surgical intervention.

## Case presentation

An 82-year-old male was admitted with complaints of retrosternal chest pain radiating to the arm. The patient was a non-smoker with no history of any comorbidities. He was diagnosed with non-ST elevation myocardial infarction. The patient was treated with dual antiplatelets (aspirin 325 mg and clopidogrel 300 mg), and low molecular weight heparin. During his hospital stay, the patient developed atrial fibrillation with a fast ventricular rate, which reverted to sinus rhythm with injectable amiodarone. He was subjected to coronary angiography via right radial arterial access (single direct anterior wall puncture), using a micropuncture access kit (Terumo), and six French sheaths were secured in place. Angiography revealed a significant left anterior descending (LAD) artery stenosis with calcification and an occluded left circumflex (LCX) artery. The procedure was performed using 3000 units of heparin for anticoagulation. Hemostasis was achieved upon completion of the procedure using a transradial band (TR band) (Terumo Medical Corporation) with 15 cc of air for compression. The TR band was deflated according to the protocol and removed over the next 40 min. No ecchymosis or hematoma was noted upon removal of the TR Band. The patient was discharged with advice to take Apixaban 2.5 mg twice daily, along with the antiplatelets.

Two days after angiography, the patient noted a small swelling over the radial puncture site that grew progressively. The patient visited for consultation after eight days.

**Figure 1:**
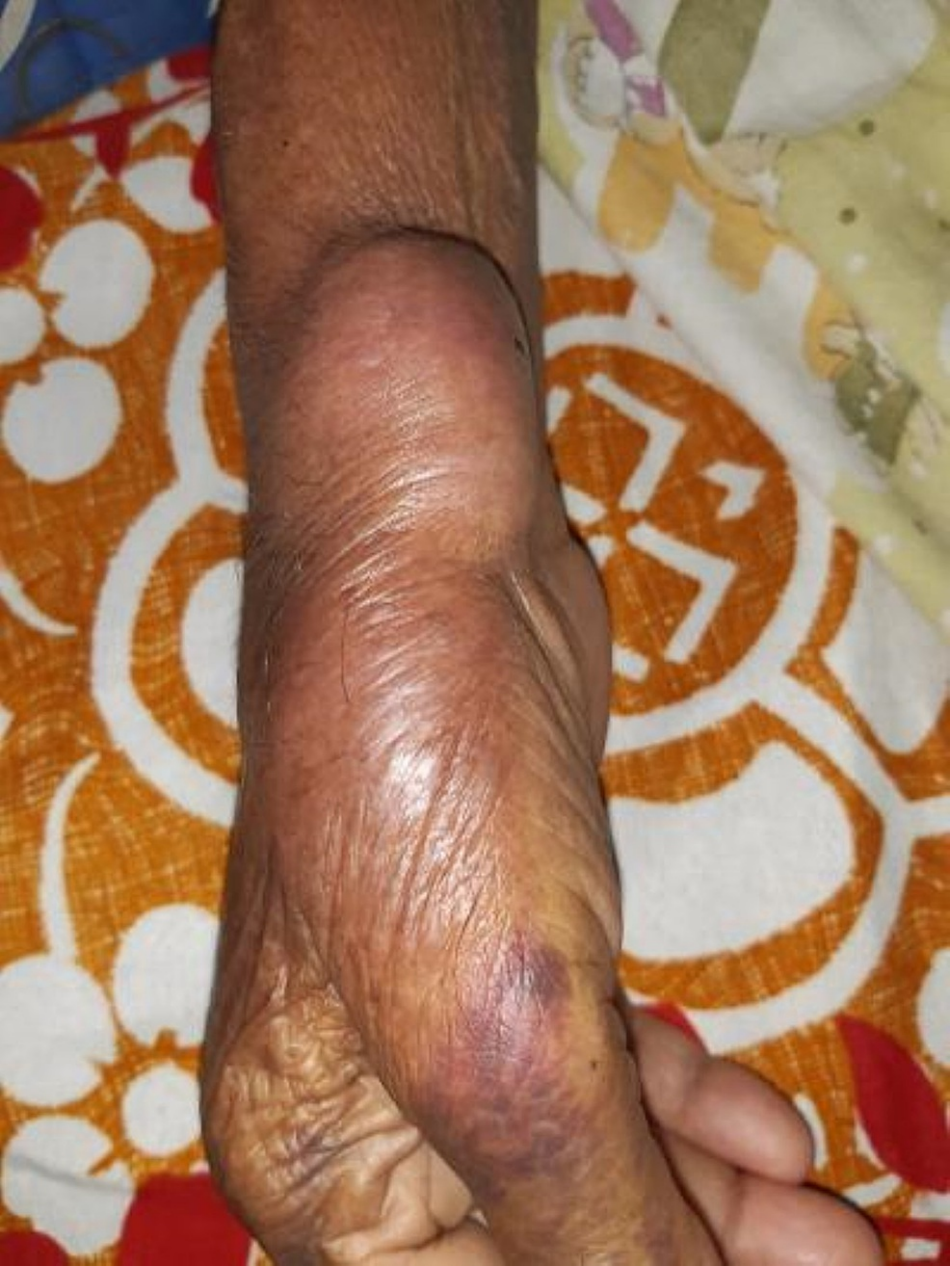
Radial pseudoaneurysm

Physical examination revealed a soft, tender, non-pulsatile swelling in the distal forearm over the right radial puncture site. The size of the swelling was 3×4 cm (Figure 1). The radial pulse, sensation distal to the puncture site, distal capillary refill, and movement of the wrist were intact. No appreciable bruit was detected. Ecchymotic patches were noted on the forearm. A provisional diagnosis of radial pseudoaneurysm was made.

**Figure 2:**
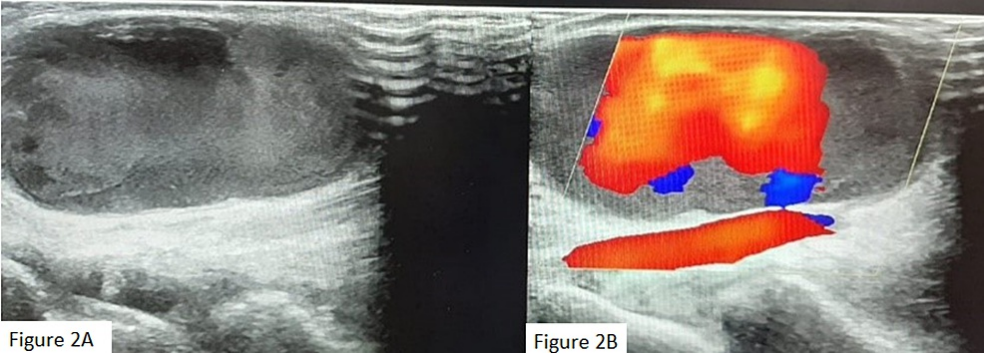
A and B, Radiological image of pseudoaneurysm. Figure 2A: Greyscale ultrasound showing an aeurysmal sac. Figure 2B: Color imaging showed a small neck from the pseudoaneurysm communicating with the radial artery.

Greyscale sonography revealed a pulsatile, thin-walled, anechoic, saccular lesion of approximately 3.4×2.2 cm. The lesion was located on the lateral aspect of the wrist with a communicating track from the right radial artery and a transverse dimension of 0.08 cm (Figure 2). Adjacent soft-tissue edema was also evident, although the right radial artery exhibited a normal flow velocity pattern.

**Figure 3:**
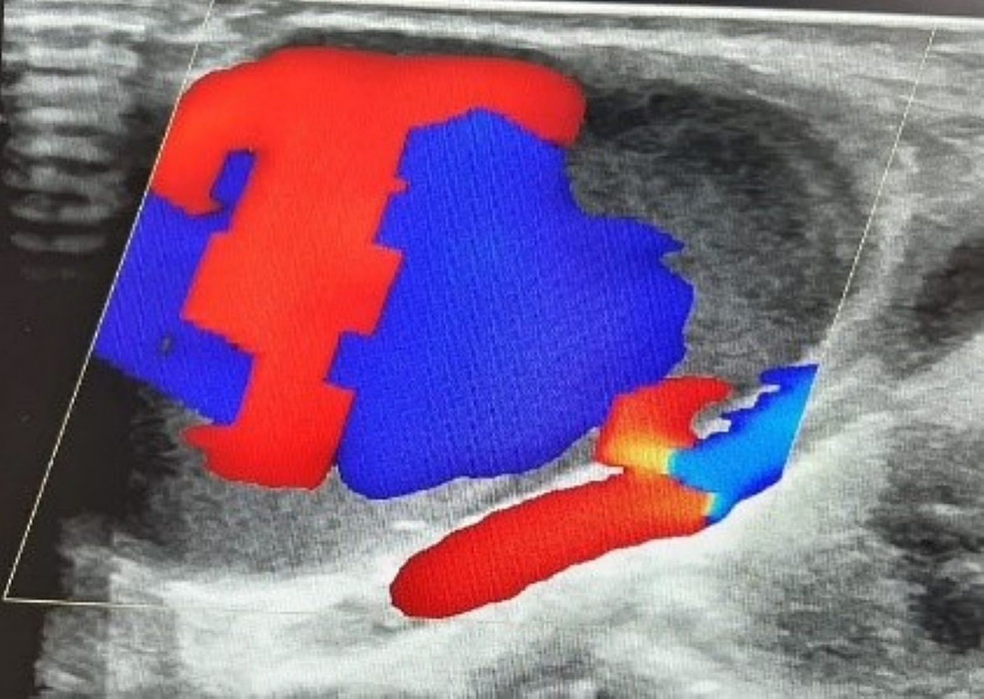
The “yin-yang” sign. Doppler ultrasound demonstrates the “yin-yang” sign, which indicates bidirectional flow due to blood swirling within the aneurysmal sac.

Color Doppler imaging demonstrated a turbulent and swirling blood flow pattern within the lesion. A portion of the turbulent flow was redirected into the aneurysmal sac at every arterial pulsation. This to-and-fro waveform pattern within the arterial lesion is known as the “yin-yang” sign (Figure 3). 

Probe compression of the pseudoaneurysm neck was performed using a linear probe of 7 Mhz frequency for one hour. This resulted in partial thrombosis of the pseudoaneurysmal sac. However, the communicating track to the radial artery was still patent. Compression was repeated for another 45 min, but the closure of the aneurysmal neck was not achieved. Therefore, the surgical team decided to surgically close the aneurysm neck.

**Figure 4:**
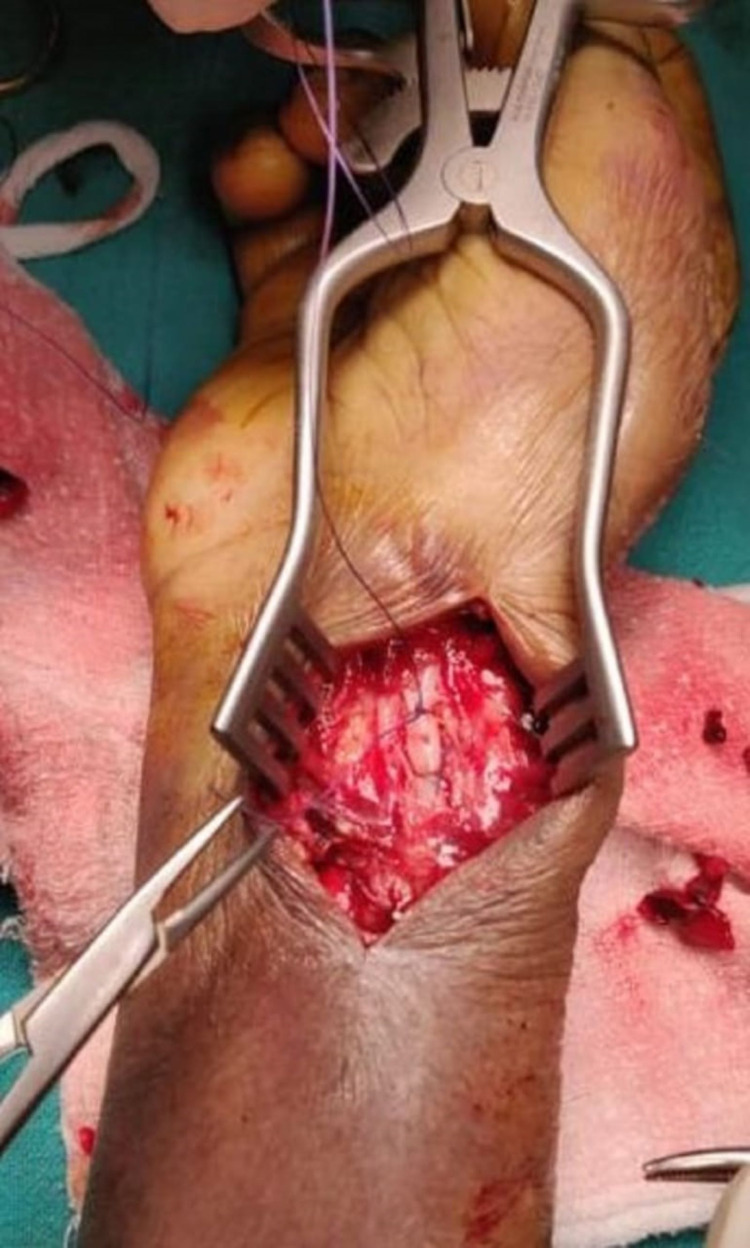
Surgical view of the ligated radial artery.

Because of the age and associated cardiac risk of the patient, the surgery was performed under a brachial plexus block with an arm tourniquet control. A longitudinal incision was made directly over the wrist swelling. The skin-deep incision revealed a cyst filled with clotted blood. Fluids and clots were removed, and the fascial tissue was dissected to isolate the radial artery. The dissection revealed a tear on the anterior surface of the radial artery that communicated with the surrounding hematoma. The artery was thick-walled and calcified. The arterial tear was closed with a Prolene suture. Because of the calcified appearance of the artery, both the proximal and distal sections of the radial artery were ligated to prevent a recurrence of the leakage (Figure 4).

**Figure 5:**
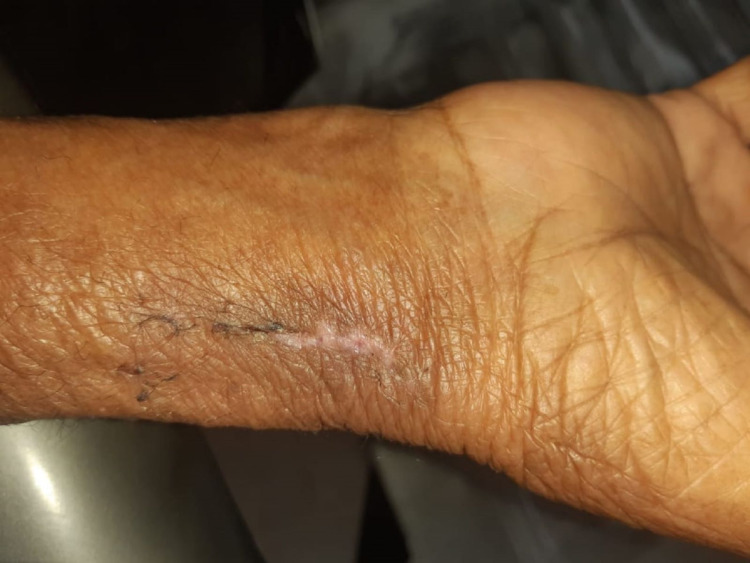
Healed wound after removal of the sutures.

The hematoma was cleared from the adjoining forearm area that may have developed after the probe compression. After the tourniquet was released, hemostasis was achieved, and the wound was closed with a tube drain inside. The drain was removed after 48 hours, and no further swelling or wound complications developed. No distal neurovascular deficit was noted in the hand. Skin sutures were removed after two weeks (Figure 5).

## Discussion

A pseudoaneurysm is an accumulation of blood outside the artery within the surrounding parenchymal tissue. In contrast to a true aneurysm, which is lined with three arterial wall layers (intima, media, and adventitia), a pseudoaneurysm is lined with fibrous scar tissue. Pseudoaneurysms are usually caused by arterial wall lacerations during trauma or cannulation. The radial artery is an uncommon site for pseudoaneurysm formation [7]. The incidence of pseudoaneurysm is only about 0.05% after catheterization of the radial artery [8-10]. The factors associated with this complication include repeated arterial puncture attempts and catheter infection [10]. Other predisposing factors include advanced age, longer duration of catheterization, large sheath diameter, anticoagulant/antiplatelet use, coagulation disorders, and incomplete hemostasis.

In contrast to a simple hematoma, a pseudoaneurysm may be pulsatile or carry an audible bruit. Enlarged pseudoaneurysms can cause mass effect, digital ischemia, or symptoms of nerve compression. The increased swelling caused pain in our patient. Compression of the veins and distal embolization from microemboli are rare complications of pseudoaneurysms [11]. For arterial pseudoaneurysms, rupture due to the thin fibrotic layer covering the aneurysm is also a risk [12]. In our patient, the pseudoaneurysmal sac was enlarged, and the communication with the radial artery did not successfully thrombose after moderate probe compression. Hence, surgical intervention was needed.

Ultrasound is the most readily available imaging modality to diagnose a pseudoaneurysm and its associated arterial wall defect. Variable echogenicity inside the hematoma indicates fluctuations in blood flow. Color flow Doppler demonstrates a swirling and turbulent flow. The important pathognomonic sign is a to-and-fro waveform within the arterial lesion on spectral Doppler imaging, known as the “yin-yang” sign [13].

Ultrasound helps to differentiate a pseudoaneurysm from an abscess or simple cyst. An abscess usually exhibits an irregular thickened wall, internal debris with posterior acoustic enhancement, and a positive “squish sign” (movement of abscess debris) on probe compression. A cyst often appears as a smooth, well-circumscribed circular structure with anechoic contents. However, neither of these lesions exhibits a consistent swirling flow pattern on color Doppler.

Radial pseudoaneurysm management is aimed at discontinuing the communication between the artery and the hematoma and repair of the wall lesion. Treatment usually depends on the location, symptoms, presence of thrombi, etiology, distal circulation, and collateral formation. Small (< 3 cm), non-enlarging, asymptomatic pseudoaneurysms are usually only monitored because most will thrombose spontaneously within four weeks [14]. Ultrasound-guided compression can be used to occlude the neck of the pseudoaneurysm. Ultrasound-guided thrombin injection can also be used to convert fibrinogen into fibrin, leading to clot formation. This method is used more commonly for the treatment of femoral artery pseudoaneurysms [15-17], but a few successful cases for the radial artery have been reported [18-19].

Surgical management is usually recommended in patients with large, expanding, symptomatic, or infected pseudoaneurysms, or pseudoaneurysms that failed initial conservative treatment. These pseudoaneurysms are at a greater risk of thromboembolism and rupture.

Our patient was on dual antiplatelet. An anticoagulant was added in view of atrial fibrillation. No complication was noted on the day of cardiac catheterization. Later on, he developed a large pseudoaneurysm at the radial artery puncture site, probably due to triple blood thinners and calcified artery. He underwent surgical management due to the size of the hematoma, worsening symptoms, and failed probe compression. Fortunately, the surgery was free of complications, and the patient recovered without any complaints at the follow-up visit. He preferred to be on optimum medical management for his cardiac lesion.

## Conclusions

The increasing use of radial artery catheterization for coronary angiography and percutaneous coronary intervention will probably lead to more procedural complications. Radial pseudoaneurysms are rare but potentially harmful complications. Identifying potential high-risk patients, ensuring a more prolonged compression in these patients, and close monitoring even after removing the compression bandage/hemostatic device are crucial for the prevention and early identification of this complication.
